# Short term synaptic depression improves information transfer in perceptual multistability

**DOI:** 10.3389/fncom.2013.00085

**Published:** 2013-07-01

**Authors:** Zachary P. Kilpatrick

**Affiliations:** Department of Mathematics, University of HoustonHouston, TX, USA

**Keywords:** binocular rivalry, neural field, ring model, bump attractor, short term depression

## Abstract

Competitive neural networks are often used to model the dynamics of perceptual bistability. Switching between percepts can occur through fluctuations and/or a slow adaptive process. Here, we analyze switching statistics in competitive networks with short term synaptic depression and noise. We start by analyzing a ring model that yields spatially structured solutions and complement this with a study of a space-free network whose populations are coupled with mutual inhibition. Dominance times arising from depression driven switching can be approximated using a separation of timescales in the ring and space-free model. For purely noise-driven switching, we derive approximate energy functions to justify how dominance times are exponentially related to input strength. We also show that a combination of depression and noise generates realistic distributions of dominance times. Unimodal functions of dominance times are more easily told apart by sampling, so switches induced by synaptic depression induced provide more information about stimuli than noise-driven switching. Finally, we analyze a competitive network model of perceptual tristability, showing depression generates a history-dependence in dominance switching.

## Introduction

Ambiguous sensory stimuli with two interpretations can produce perceptual rivalry (Blake and Logothetis, 2002). For instance, presenting two orthogonal gratings to either eye results in perception switching between gratings repetitively—binocular rivalry (Leopold and Logothetis, 1996). Perceptual rivalry can also be triggered by a single stimulus with two interpretations, like the Necker cube (Orbach et al., 1963). The switching process in perceptual rivalry is considerably stochastic—a histogram of the dominance times of each percept spreads across a broad range (Fox and Herrmann, 1967). Senses other than vision also exhibit perceptual rivalry. When two different odorants are presented to the two nostrils, a similar phenomenon occurs with olfaction, termed “binaral” rivalry (Zhou and Chen, 2009). Similar experiences have been evoked in the auditory (Deutsch, 1974; Pressnitzer and Hupé, 2006) and tactile (Carter et al., 2008) system.

Several principles govern the relationship between the strength of ambiguous stimuli and the mean switching statistics in perceptual rivalry (Levelt, 1965). “Levelt's propositions” relate stimulus contrast to the *mean dominance times*: (1) increasing the contrast of one stimulus increases the proportion of time that stimulus is dominant; (2) increasing the contrast of one stimulus does not affect its average dominance time; (3) increasing the contrast of one stimulus increases the rivalry alternation rate; and (4) increasing the contrast of both stimuli increases the rivalry alternation rate. Properties of the input also affect the stochastic variation in the dominance times (Brascamp et al., 2006). For instance, a histogram of dominance times is well fit by a gamma distribution (Fox and Herrmann, 1967; Lehky, 1995; van Ee, 2009). The fact that dominance times are not exponentially distributed suggests some background slow adaptive process plays a role in providing a non-zero peak in the dominance histograms (Shpiro et al., 2009). Two commonly proposed mechanisms for this adaptation are spike frequency adaptation and short term synaptic depression (Laing and Chow, 2002; Wilson, 2003; Shpiro et al., 2007). A stronger case can be made for the existence of adaptation in perceptual processing networks by examining results of experiments on *perceptual tristability* (Hupe, 2010). Here, perception alternates between three possible choices and subsequent switches are determined by the previous switch (Naber et al., 2010). This memory suggests switches in perceptual multistability are not purely noise-driven (Moreno-Bote et al., 2007).

Most theoretical models of perceptual rivalry employ two pools of neurons, each selective to one percept, coupled to one another by mutual inhibition (Matsuoka, 1984; Laing and Chow, 2002; Shpiro et al., 2007; Seely and Chow, 2011). With no other mechanisms at work, such architectures lead to *winner-take-all* states, where one pool of neurons inhibits the other indefinitely (Wang and Rinzel, 1992). However, switches between the dominance of one pool and the other can be initiated with the inclusion of fluctuations (Moreno-Bote et al., 2007) or an adaptive process (Laing and Chow, 2002; Shpiro et al., 2007). Combining the two mechanisms leads to dominance times that are distributed according to the gamma distribution (Laing and Chow, 2002; Shpiro et al., 2009; van Ee, 2009). Thus, slow adaptation and noise allow sampling of the stimulus through changes in network activity.

In light of these observations, we wish to consider the role adaptive mechanisms play in properly sampling ambiguous stimuli in a mutual inhibitory network. Two stimuli of different orientations are presented to the network (Levelt, 1965). The network outputs a time-dependent, orientation-dependent firing rate, whose peak switches between two locations determined by the two stimuli. We think of the information output by the network as a series of dominance times. We will study how well the relative strength of the two stimuli (information) is encoded by the amount of time each subpopulation remains active during a dominance period (Levelt, 1965; Moreno-Bote et al., 2007). Purely fluctuation driven switching provides a noisy sample of the two percepts, but adaptation driven switching provide an extremely reliable sampling of percept contrast (Shpiro et al., 2009). As the level of adaptation is increased and noise is decreased, mutual inhibitory networks encode information about ambiguous stimuli better. We focus specifically on the adaptive mechanism of short term synaptic depression (Tsodyks and Markram, 1997).

Using parameterized models, we will explore how synaptic depression improves the ability of a network to extract stimulus contrasts. First, we study how much information can be determined about the contrast of each of the two percepts of an ambiguous stimulus. In the case of a *winner-take-all* solution, only information about a single percept can be known, since the pool of neurons encoding the other percept is quiescent. We will study this using an anatomically motivated neural field model of an orientation column with synaptic depression (York and van Rossum, 2009; Kilpatrick and Bressloff, 2010a). Increasing the strength of synaptic depression leads to a bifurcation which produces rivalrous oscillations. When rivalrous switching occurs through a combination of depression and noise, we show stronger depression improves the transfer of information. We also analyze a reduced network model with depression and noise to help study the combined effects of noise and depression on perceptual switching. Finally, we study perceptual tristability as oscillations generated in a three population network, where each population spends time in dominance. This shows depression generates a history dependence in switching that would not arise in the network with purely noise-driven switching.

## Materials and methods

### Ring model with synaptic depression

As a starting point, we consider a model for processing the orientation of visual stimuli (Ben-Yishai et al., 1995; Bressloff and Cowan, 2002) which also includes short term synaptic depression (York and van Rossum, 2009; Kilpatrick and Bressloff, 2010a). Since GABAergic inhibition is much faster than AMPA-mediated excitation (Kawaguchi and Kubota, 1997), we assume that inhibition is slaved to excitation as in Amari (1977). Reduction this disynaptic pathway and assuming depression acts on excitation (Tsodyks and Markram, 1997), we then have the model

(1a) $$\tau_{m}\dot{u}=-u(x,t)+w*(qf(u))+I(x)+\xi (x,t),$$

(1b) $$\tau \dot{q}=1-q(x,t)-\beta q(x,t)f(u(x,t)).$$

Here *u*(*x, t*) measures the synaptic input to the neural population with stimulus preference *x* ∈ [−π/2, π/2] at time *t*, evolving on the timescale τ_*m*_. Synaptic interactions are described by the integral term

$$w*(qf(u))=\int_{-\pi /2}^{\pi /2}w(x-y)q(y,t)f(u(y,t))\text{dy},$$

so *w*(*x* − *y*) describes the strength (amplitude of *w*) and net polarity (sign of *w*) of synaptic interactions from neurons with stimulus preference *y* to those with preference *x*. The modulation of the synaptic strength is given by the cosine

(2) w(x−y)=cos(2(x−y)),

so neurons with similar orientation preference excite one another and those with dissimilar orientation preference disynaptically inhibit one another (Ben-Yishai et al., 1995; Ferster and Miller, 2000). The factor *q*(*x, t*) measures of the fraction of available presynaptic resources, which are depleted at a rate β*f* (Tsodyks and Markram, 1997), and are recovered on a timescale specified by the time constant τ (Chance et al., 1998). Firing rates are given by taking the gain function *f*(*u*) of the synaptic input, which we usually proscribe to be (Wilson and Cowan, 1973)

(3) $$f(u)=\frac{1}{1+{\text{e}}^{-\gamma (u-\kappa )}},$$

and often take the γ → ∞, so (Amari, 1977)

(4) $$f(u)=H(u-\kappa )=\{\begin{array}{ll} 0 & :u<\kappa ,\\ 1 & :u\geq \kappa . \end{array}$$

External input, representing flow from upstream in the visual system is prescribed by the time-independent function *I*(*x*) (Ben-Yishai et al., 1995; Bressloff and Cowan, 2002). For the majority of our study of Equation (1), we employ the bimodal stimulus

(5) $$I(x)=-I_{0}\cos(4x)+I_{a}\sin(2x),$$

representing stimuli at the two orthogonal angles −π/4 and π/4 and *I*_0_ controls the mean of each peak and *I*_*a*_ controls the level of asymmetry between the peaks. Effects of noise are described by the stochastic process 〈ξ(*x, t*)〉 with 〈ξ(*x, t*)〉 = 0 and 〈ξ(*x, t*)ξ(*y, s*)〉 = *C*(*x − y*)δ(*t − s*), and spatial correlations are take to have a cosine profile *C*(*x*) = π cos(*x*).

We assume units of time *t* to be 10 ms each. Excitatory synaptic time constants are roughly 10 ms (Häusser and Roth, 1997), so we set τ_*m*_ = 1 (10 ms). Experimental observations have shown synaptic resources specified *q* are recovered on a timescale of 200–800 ms (Tsodyks and Markram, 1997), so we require τ is between 20 and 80, usually setting it to be τ = 50. Our parameter β can then be varied independently to adjust the effective depletion rate of synaptic depression. In our numerical simulations, we typically use the winner-take-all state as the initial condition.

### Idealized competitive neural network

We also study space-free competitive neural networks with synaptic depression (Shpiro et al., 2007). As a general model of networks connected by mutual inhibition, we consider the system (Laing and Chow, 2002; Moreno-Bote et al., 2007; Shpiro et al., 2007)

(6a) $${\dot{u}}_{R}=-u_{R}(t)+f(I_{R}-q_{L}(t)u_{L}(t))+\xi_{1}(t),$$

(6b) $${\dot{u}}_{L}=-u_{L}(t)+f(I_{L}-q_{R}(t)u_{R}(t))+\xi_{2}(t),$$

(6c) $$\tau {\dot{q}}_{R}=1-q_{R}(t)-\beta u_{R}(t)q_{R}(t),$$

(6d) $$\tau {\dot{q}}_{L}=1-q_{L}(t)-\beta u_{L}(t)q_{L}(t),$$

where *u*_*j*_(*t*) represents the firing rate of the *j* = *L, R* population. The resource usage rate by synapse projecting from population *j* = *L, R* is specified by β*u*_*j*_*q*_*j*_ and the resource recovery timescale is τ. Fluctuations are introduced into population *j* with the independent white noise processes ξ_*j*_ with 〈*x*_*j*_ (*t*)〉 = 0 and 〈ξ_*j*_(*t*)ξ_*j*_(*s*)〉 = εδ(*t − s*). Units of time are taken to be 10 ms each. In numerical simulations, *u*_*j*_(0) are initialized by randomly drawing from a uniform distribution on [0, 1]; *q*_*j*_(0) are initialized by randomly drawing from a uniform distribution on [1/(1 + β), 1].

### Numerical simulation of stochastic differential equations

The spatially extended model (Equation 1) is simulated using an Euler–Maruyama method with a timestep dt = 10^−4^, using Riemann integration on the convolution term with 2000 spatial grid points. A population is considered dominant if the peak of its activity bump is higher than the other; switches occur when the other bump attains a higher peak. The reduced network (Equation 6) was also simulated using Euler–Maruyama with a timestep dt = 10^−6^. Population *j* is considered dominant when *u*_*j*_ > *u*_*k*_ (*j* ≠ *k*); switches occur when the inequality switches direction. To generate histograms of dominance times, we simulated systems for 20,000s.

### Fitting dominance time distributions

To generate the theoretical curves presented for exponentially distributed dominance times, we simply take the mean of dominance times and use it as the scaling in the exponential (Equation 28). For those densities that we presume are gamma distributed, we solve a linear system to fit the constants *c*_1_, *c*_2_, and *c*_3_ of

(7) $$f(T)={\text{e}}^{c_{\text{1}}}T^{c_{\text{2}}}e^{-c_{\text{3}}T}$$

an alternate form of Equation (30). Upon taking the logarithm of Equation (7), we have the linear sum

(8) $$\ln f(T)=c_{1}+c_{2}\ln T-c_{3}T.$$

Then, we select three values of the numerically generated distribution *p*^*n*^(*T*^*n*^) along with its associated dominance times: (*T*^*n*^_1_, *p*^*n*^_1_); (*T*^*n*^_2_, *p*^*n*^_2_); (*T*^*n*^_3_, *p*^*n*^_3_) where *p*^*n*^_*j*_ = *p*^*n*^(*T*^*n*^_*j*_). We always choose *T*^*n*^_2_ = arg max_*T*_ *p*^*n*^(*T*) as well as *T*^*n*^_1_ = *T*^*n*^_2_/2 and *T*^*n*^_3_ = 3*T*^*n*^_2_/2. It is then straightforward to solve the linear system

$$\left(\begin{array}{ccc} 1 & \ln T_{1}^{n} & -T_{1}^{n}\\ 1 & \ln T_{2}^{n} & -T_{2}^{n}\\ 1 & \ln T_{3}^{n} & -T_{3}^{n} \end{array}\right)\left(\begin{array}{c} c_{1}\\ c_{2}\\ c_{3} \end{array}\right)=\left(\begin{array}{c} \ln p_{1}^{n}\\ \ln p_{2}^{n}\\ \ln p_{3}^{n} \end{array}\right)$$

using the\command in MATLAB.

## Results

We now present results that reveal the importance of synaptic depression in preserving information about bimodal stimuli. No previous work, to our knowledge, has studied how activity in a ring model with depression (Equation 1) can be collapsed to a low dimensional oscillation. The oscillation results from a combination of depression and mutual inhibition, which produces population dominance times and can thus be sampled to give information about the strength of the stimulus that produced them. Once noise is added to these low dimensional oscillations, dominance time distributions still remain relatively tight, which can be sampled to infer relative contrasts of each input. We contrast this with a previous cue orientation selective model which used a heterogeneous population of spiking neurons with lateral inhibition and slow adaptation, so chaos rather than noise produced apparent stochasticity in dominance times (Laing and Chow, 2002). We can use an energy function for a reduced system to approximate the relative effect of depression and noise on dominance times. These energy methods are also useful in the study of perceptual tristability, where we also show depression introduces a history dependence in dominance transitions.

### Deterministic switching in the ring model

To start we consider the ring model with depression (Equation 1) in the absence of noise, so ξ ≡ 0. In previous work, noise-free versions of Equation (1) have been analyzed to explore how synaptic depression can generate traveling pulses (York and van Rossum, 2009; Kilpatrick and Bressloff, 2010b), self-sustained oscillations (Kilpatrick and Bressloff, 2010b), and spiral waves in two-dimensions (Kilpatrick and Bressloff, 2010c). Here, we will extend previous work that explored input-driven oscillations in two-layer networks possessing statistics matching binocular rivalry (Kilpatrick and Bressloff, 2010a). We think of Equation (1) as a model of *monocular rivalry*, since oscillations can be due to competition between representations in a single orientation column (Ben-Yishai et al., 1995). Competition between ocular dominance columns (Kilpatrick and Bressloff, 2010a) is not necessary for our theory. For exposition, we will employ specific functional forms: cosine weight (Equation 2); a Heaviside firing rate function (Equation 4); and a bimodal input (Equation 5).

#### Winner take all state

**Figure 1:**
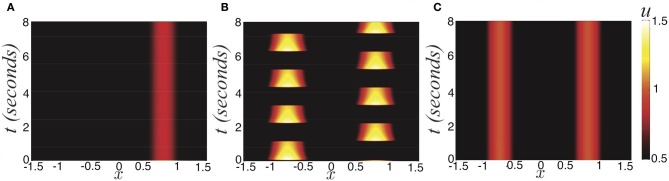
**Three possible active states of the noise-free stimulus driven ring model with depression (Equation 1). (A)** Winner take all (*I*_0_ = 0.6) defined by the bump half-width *a*; **(B)** Rivalrous oscillations (*I*_0_ = 0.84); **(C)** Fusion (*I*_0_ = 1), where initial condition is (Equation 16). Other parameters are κ = 0.5, β = 1, and τ = 50.

We now look for winner-take-all solutions, as shown in Figure 1A. These states consist of a single activity bump arising in the network, representing only one of the two percepts contained in the bimodal stimulus (Equation 5). These are stationary in time, so *u*_*t*_ = *q*_*t*_ = 0, implying *u* = *U*(*x*) and *q* = *Q*(*x*). Also, they are single bump solutions, so there is a single region *x* ∈ (π/4 − *a*, π/4 + *a*) that is superthreshold (*U*(*x*) > κ). The parameter *a* is the half-width of the bump. We assume the right stimulus is represented by a bump, although we can derive analogous results when the left stimulus is represented. The steady state solution is then determined

(9) $$\begin{array}{l} U(x)=\int_{\pi /4\;-\;a}^{\pi /4\;+\;a}\cos(2(x-y))Q(y)\text{dy}-I_{\text{0}}\cos(\text{4}x)\\ \;+I_{a}\sin(2x), \end{array}$$

(10) $$Q(x)={\left[1+\beta H(U(x)-\kappa )\right]}^{-1},$$

so by plugging Equation (10) into (9) and using cos (2(*x − y*)) = cos(2*x*) cos(2*y*) + sin(2*x*) sin(2*y*) we have

$$U(x)=A\cos(2x)+(B+I_{a})\sin(2x)-I_{0}\cos(4x),$$

where the constants *A, B* can be computed

$$\begin{array}{l} A=\frac{1}{1+\beta }\int_{\pi /4\;-\;a}^{\pi /4\;+\;a}\cos(2x)\text{d}x\text{=0},\\ B=\frac{1}{1+\beta }\int_{\pi /4\;-\;a}^{\pi /4\;+\;a}\sin(2x)\text{d}x\text{=}\frac{\sin(\text{2}a)}{\text{1+}\beta }. \end{array}$$

Therefore, by simplifying the threshold condition, *U*(π/4 ± *a*) = κ, we have

(11) $$U(\pi /4\pm a)=\frac{\sin(4a)}{2(1+\beta )}+I_{0}\cos(4a)+I_{a}\cos(2a)=\kappa .$$

The implicit Equation (11) can be solved numerically using root finding algorithms. For symmetric inputs (*I*_*a*_ = 0), we can solve (Equation 11) explicitly

(12) $$a=\frac{1}{2}\tan^{-1}\left[\frac{1\pm \sqrt{1+4{\left(1+\beta \right)}^{2}(I_{0}^{2}-\kappa^{2})}}{2(1+\beta )(I_{0}+\kappa )}\right],$$

and winner-take-all solutions take the form

(13) $$U(x)=\frac{\sin(2a)}{1+\beta }\sin(2x)-I_{0}\cos(4x)+I_{a}\sin(2x).$$

With this solution, we can relate the parameters of the model to the existence of the winner-take-all state. To do so, we need to look at a second condition that must be satisfied, *U*(*x*) < κ for all *x* ∉ (π/4 − *a*, π/4 + *a*). Since the function (Equation 13) is bimodal across (−π/2, π/2), we check the other possible local maximum at *x* = −π/4 as

(14) $$U(\pi /4)=I_{0}-I_{a}-\frac{\sin(2a)}{1+\beta }<\kappa .$$

At the point in parameter space where the Equation (14) is violated, a bifurcation occurs, so the winner-take-all state ceases to exist. This surface in parameter space is given by the equation

(15) $$I_{0}=\kappa +I_{a}+\frac{\sin(2a)}{1+\beta },$$

along with the explicit formula for the bump half-width (Equation 12). Beyond the bifurcation boundary (Equation 15), one of two behaviors can occur. Either there is a symmetric two-bump solution that exists, the fusion state (Wolfe, 1986; Blake, 1989; Shpiro et al., 2007), or rivalrous oscillations (Levelt, 1965; Blake and Logothetis, 2002).

#### Fusion state

Experiments on ambiguous stimuli have shown sufficiently strong contrast rivalrous stimuli can be perceived as a single fused image (Blake, 1989; Buckthought et al., 2008). This should not be surprising, considering stereoscopic vision and audition behave in exactly this way (Wolfe, 1986). However, the contrast necessary to evoke this state with dissimilar images is much higher than with similar images (Blake and Logothetis, 2002). The fusion state (Figure 1C) is represented as two disjoint bumps. Therefore

$$\begin{array}{l} U(x)=\frac{1}{1+\beta }\left[\int_{-\pi /4-b}^{-\pi /4+b}+\int_{\pi /4-a}^{\pi /4+a}\right]\cos(2(x-y))\text{dy}\\ \;-I_{0}\cos(4x)+I_{a}\sin(2x). \end{array}$$

Computing the integrals, we find 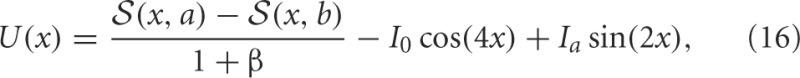 where 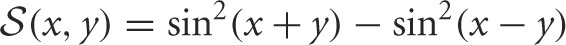. Requiring the threshold conditions *U*(−π/4 ± *b*) = *U*(π/4 ± *a*) = κ are satisfied, 
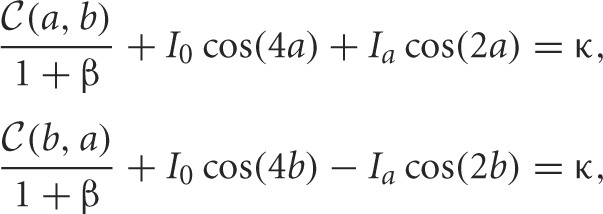
 where 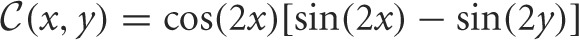, which implicitly relates parameters to the half-widths *a, b* of each bump. We will now study rivalrous oscillations by simply constructing them using a fast-slow analysis.

#### Rivalrous oscillations

**Figure 2:**
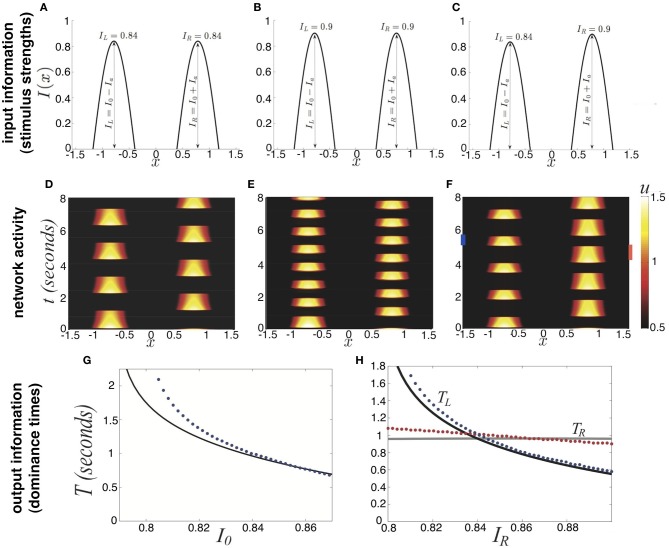
**Dependence of rivalry dominance times on the amplitudes of the bimodal input (Equation 5). (A–C)** Various profiles of the external input *I*(*x*), showing only positive part. Increasing *I*_0_ increases both peaks; increasing *I*_*a*_ decreases the left and increases the right peak. **(D–F)** Rivalrous oscillations in the neural activity *u*(*x, t*) corresponding to the input in **(A–C)**. Dominance times decrease from **(D)** to **(E)** since the input amplitude increases from **(A)** to **(B)**. **(F)** Dominance time of right input (red bar : *T*_*R*_ ≈ 0.9 s) is longer than left (blue bar : *T*_*L*_ ≈ 0.6 s) for asymmetric input in **(C)**. **(G)** Increasing the strength of the symmetric (*I*_*a*_ = 0) bimodal input (Equation 5) decreases the dominance time *T* of both populations. Our theory (black) computed from fast-slow analysis (Equation 19) fits results of numerical simulations (blue) well. **(H)** For asymmetric inputs (*I*_*a*_ ≠ 0), we find that varying *I*_*R*_ = *I*_0_ + *I*_*a*_ while keeping *I*_*L*_ = *I*_0_ − *I*_*a*_ fixed changes the dominance times of the left percept *T*_*L*_ (blue) much more than that of the right percept *T*_*R*_ (red). Other parameters are κ = 0.5, β = 1, and τ = 50.

Oscillations can occur, where the two bump locations trade dominance successively (Figure 1B). We will show Levelt's proposition (i) holds; increasing the contrast of a stimulus (Figures 2A–C) increases the proportion of time that stimulus is dominant (Figures 2D–F). This information is not revealed when the system is stuck in a winner-take-all state. Thus, synaptic depression can unmask otherwise hidden stimuli. We will also examine how well the noise-free version of Equation (1) recapitulates Levelt's other propositions concerning the mean dominance of percepts.

To study oscillations, we assume that the timescale of synaptic depression τ » τ_*m*_, is long enough that we can decompose (Equation 1), with ξ ≡ 0, into a fast and slow system (Laing and Chow, 2002; Kilpatrick and Bressloff, 2010a). Synaptic input *u* then tracks the slowly varying state of the synaptic scaling term *q*. We have also verified in simulations *q* is essentially piecewise constant in space, in the case of the Heaviside non-linearity (Equation 4), which yields

(17) $$\begin{array}{l} u(x,t)\approx \int_{-\pi /2}^{\pi /2}\cos(2(x-y))q(y,t)H(u(y,t)-\kappa )\text{d}y\\ \;-I_{0}\cos(4x), \end{array}$$

and *q* is governed by Equation (1b). To start, we will also assume a symmetric bimodal input (*I*_*a*_ = 0). This way, we can simply track *q* in the interior of one of the bumps, given *q*_*i*_(*t*) = *q*(π/4, *t*). Solving the resulting piecewise system of differential equations, we can derive an implicit formula for

(18) $$q_{0}=\frac{1}{1+\beta }+\frac{\beta }{1+\beta }{\text{e}}^{\text{−T/}\tau }\text{−}({\text{1−q}}_{\text{0}}){\text{e}}^{\text{−2T/}\tau },$$

the value of the synaptic depression variable inside a bump just prior to a switch. We can rearrange (Equation 18) to yield a formula for the dominance time

(19) $$T=\tau \ln\left[\frac{\beta +\sqrt{\beta^{2}-4(1+\beta )(1-q_{0})[(1+\beta )q_{0}-1]}}{2(1+\beta )q_{0}-2}\right],$$

so that we now must specify the value *q*_0_. We can examine the fast Equation (17), solving for the form of the slowly narrowing right bump during its dominance phase

(20) $$\begin{array}{l} u(x,t)=q_{i}(t)\left[\sin^{2}(x+a(t))-\sin^{2}(x-a(t))\right]\\ \;-\;I_{0}\cos(4x). \end{array}$$

We solve for the slowly changing width *a*(*t*) by enforcing the threshold condition *u*(π/4 ± *a*(*t*), *t*) = κ and using trigonometric identities to find

(21) $$a(t)=\frac{1}{2}\tan^{-1}\left[\frac{q_{i}(t)+\sqrt{q_{i}{\left(t\right)}^{2}+4(I_{0}^{2}-\kappa^{2})}}{2(I_{0}+\kappa )}\right].$$

We can also identify the maximal value of *q*_*i*_(*t*) = *q*_0_ which still leads to the right bump suppressing the left. Once *q*_*i*_(*t*) falls below *q*_0_, the other bump escapes suppression, flipping the dominance of the current bump. This is the point at which the other bump of Equation (20) rises above threshold, as defined by the equation *I*_0_ − *q*_0_ sin(2*a*_0_) = κ. Combining this with Equation (21) and solving the resulting algebraic equation, we find

(22) $$q_{0}=\frac{2I_{0}\sqrt{\left(I_{0}-\kappa )(3I_{0}+\kappa \right)}}{3I_{0}+\kappa }.$$

**Figure 3:**
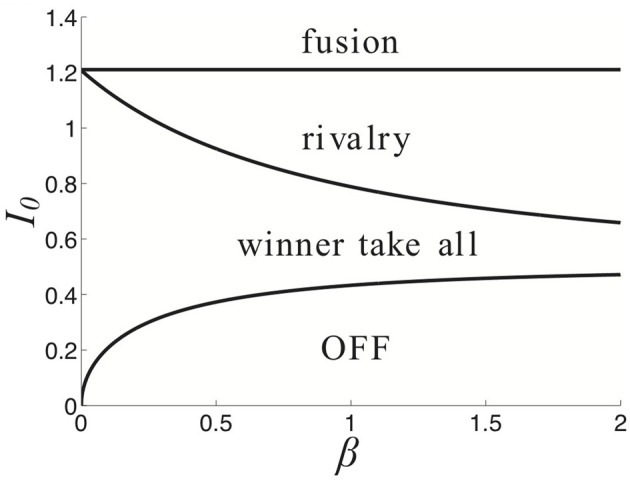
**Partition of parameter space (β, *I*_0_) into various stimulus-induced states of (Equation 1) when ξ ≡ 0, κ = 0.5, and τ = 50**.

The amplitude of synaptic depression is excluded from Equation (22), but we know *q*_0_ ∈ ([1 + β]^−1^, 1). This establishes a bounded region of parameter space in which we can expect to find rivalrous oscillations, which we use to construct a partitioning of parameter space in Figure 3. We can also now approximate the dominance time using Equation (19) with (22), as shown in Figure 2G.

In the case of an asymmetric bimodal input (*I*_*a*_ > 0), we can also solve for explicit approximations to the dominance times of the right *T*_*R*_ and left *T*_*L*_ populations. Following the same formalism as for the symmetric input case

(23) $$T_{R}=\tau \ln\left[\frac{Q_{+}+\sqrt{Q_{+}^{2}-B_{R}}}{2(1+\beta )q_{R}-2}\right],$$

(24) $$T_{L}=\tau \ln\left[\frac{Q_{-}+\sqrt{Q_{-}^{2}-B_{L}}}{2(1+\beta )q_{L}-2}\right],$$

where *Q*_±_ = β ± (1 + β) (*q*_*R*_ − *q*_*L*_) and *B*_*R, L*_ = 4(1 + β) (1 − *q*_*L, R*_)[(1 + β)*q*_*R, L*_ − 1], in terms of the local values *q*_*L*_ and *q*_*R*_ of the synaptic scaling in the right and left bump immediately prior to their suppression. Notice when *q*_*L*_ = *q*_*R*_, then *q*_*d*_ = 0 and Equations (23) and (24) reduce to Equation (19). We now need to examine the fast Equation (17) to identify these two values. This is done by generating two implicit equations for the half-width of the right bump *a*_*R*_ and *q*_*R*_ at the time of a switch

$$\begin{array}{l} \frac{q_{R}}{2}\sin(4a_{R})+I_{0}\cos(4a_{R})+I_{a}\cos(2a_{R})=\kappa ,\\ \;I_{0}-I_{a}-q_{R}\sin(2a_{R})=\kappa , \end{array}$$

which we can solve explicitly for

$$a_{R}=\frac{1}{2}\cos^{-1}\left[\frac{\kappa }{2I_{0}}+\frac{1}{2}\right],$$

and

(25) $$q_{R}=\frac{2I_{0}(I_{L}-\kappa )}{\sqrt{\left(3I_{0}+\kappa )(I_{0}-\kappa \right)}},$$

where *I*_*L*_ = *I*_0_ − *I*_*a*_ is the strength of input to the left side of the network. Likewise, we can find the value of the synaptic scaling in the left bump immediately prior to its suppression

(26) $$q_{L}=\frac{2I_{0}(I_{R}-\kappa )}{\sqrt{\left(3I_{0}+\kappa )(I_{0}-\kappa \right)}},$$

where *I*_*R*_ = *I*_0_ + *I*_*a*_ is the strength of input to the right side of the network. Using the expressions (25) and (26) we can now compute the dominance time formulae (23) and (24), showing the relationship between inputs and dominance times in Figure 2H. Notice that all of Levelt's propositions are essentially satisfied. Changing the strength of the right stimulus *I*_*R*_ has a very weak effect on the dominance time of the right percept. Thus, dominance times obey the classic description of Levelt's second proposition (Levelt, 1965). Recent evidence does suggest this only holds at high contrast (Brascamp et al., 2006), and our study is consistent with this since inputs are high contrast here, since it lies just below a fusion state. This is characteristic of competitive networks whose switches occur via an escape mechanism (Wang and Rinzel, 1992; Shpiro et al., 2007), whereby the suppressed population comes on and overtakes the previously dominant population.

Finally, we demonstrate how the strength of a symmetric input *I*_0_ and strength of depression β lead to different behaviors of the network (Equation 1) in Figure 3. For weaker synaptic depression strength β, there is a narrower range of stimulus strengths *I*_0_ for which rivalrous oscillations exist. When synaptic depression is sufficiently strong, the range of *I*_0_ that leads to a winner-take-all state narrows. For sufficiently strong *I*_0_, increasing β leads to a network that reveals a piece of the stimulus that would otherwise be kept hidden. As we will show, synaptic depression helps the network reveal stimulus information in a way that is much more reliable than noise.

### Purely stochastic switching in the ring model

**Figure 4:**
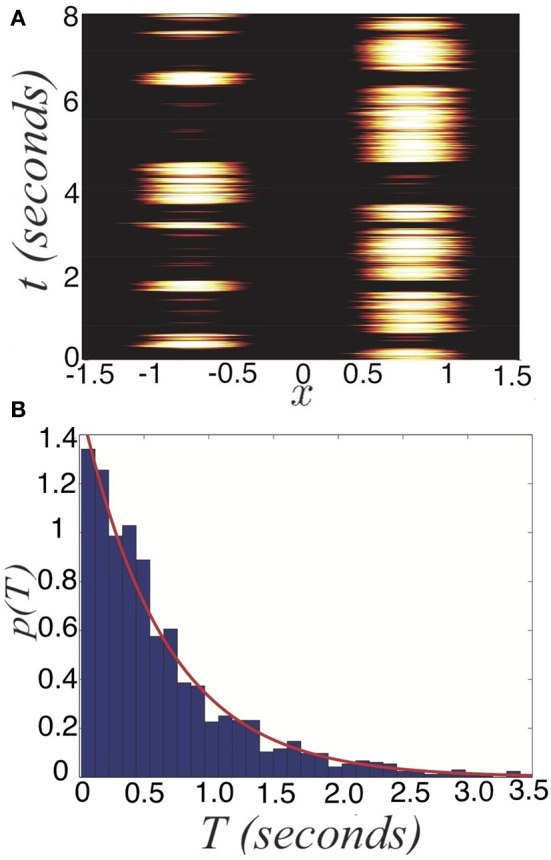
**Noise-induced switching of dominance in the depression-free ring model (Equation 27). (A)** Numerical simulations of the system for *I*_0_ = 0.9 and *I*_*a*_ = 0 in bimodal input (Equation 5). **(B)** Distribution of dominance times computed numerically (blue bars) with the exponential distribution (Equation 28) with numerically computed mean 〈*T*〉 ≈ 0.70 s (red) superimposed for *I*_0_ = 0.9. Other parameters are κ = 0.5 and ε = 0.04.

We will now study rivalrous switching brought about by fluctuations. In particular, we ignore depression and examine the noisy system

(27) $$\dot{u}(x,t)=-u(x,t)+w*f(u)+I(x)+\xi (x,t).$$

where 〈ξ(*x, t*)〉 = 0 and 〈ξ(*x, t*)ξ(*y, s*)〉 = ε*C*(*x − y*)δ(*t − s*) defines the spatiotemporal correlations of the system. Since there is no synaptic depression in the model (Equation 27), no deterministic mechanisms will generate switches between one winner-take-all state and another. Thus, consider the effects of introducing a small amount of noise (0 < ε « 1), reflective of synaptic fluctuations, with spatial correlation function *C*(*x*) = cos(*x*). Noise generates switches in between the two dominant states (Figure 4A). Activity of neurons not driven by the stimulus remains close to zero even during dominance switching. There will be no mixing of the two inputs in the networks representation of the stimulus. Dominance switching occurs via an escape mechanism (Wang and Rinzel, 1992), whereby noise drives the suppressed population on, which in turn suppresses the dominant population. As opposed to depression-induced switching, there is an exponential spread in the possible dominance times for a given set of parameters (Figure 4B). By sampling two dominance times back to back, it may be difficult to tell if the input strengths are roughly the same or not.

**Figure 5:**
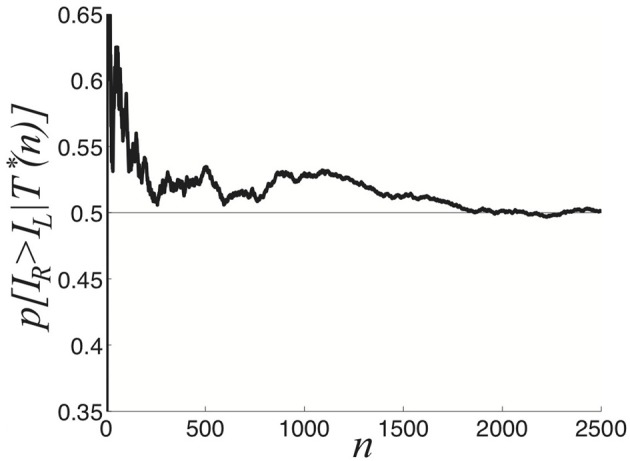
**Predicted probability right input *I*_*R*_ is higher than the left input *I*_*L*_, based on the sampling *n* cycles (2*n* switches between percepts), for symmetric inputs *I*_*L*_ = *I*_*R*_ = 0.9**. After 2000 cycles, *p*[*I*_*R*_ > *I*_*L*_ | T^*^(*n*)] ≈ 0.5. Other parameters are κ = 0.5 and ε = 0.04.

We now explore the task of discerning the relative contrasts of the two stimuli *I*_*R*_ and *I*_*L*_ based on samples of the dominance time distributions. Notice in Figure 5 that the likelihood assigned to *I*_*R*_ > *I*_*L*_ approaches 1/2 as the number of observations *n* increases. We compute *p*[*I*_*R*_ > *I*_*L*_ | T^*^(*n*)], the predicted probability *I*_*R*_ > *I*_*L*_ based on sampling dominance time pairs from *n* cycles T^*^(*n*) = {*T*^(1)^_*R*_, *T*^(1)^_*L*_; *T*^(2)^_*R*_, *T*^(2)^_*L*_; …; *T*^(*n*)^_*R*_, *T*^(*n*)^_*L*_}. As *n* → ∞, the exponential distributions approximately defining the identical probability densities *p*_*R*_(*T*_*R*_) = *p*_*L*_(*T*_*L*_) = *p*(*T*) are fully sampled and *p*(*I*_*R*_ > *I*_*L*_ | T^*^(∞)) = 1/2, as in Figure 5.

**Figure 6:**
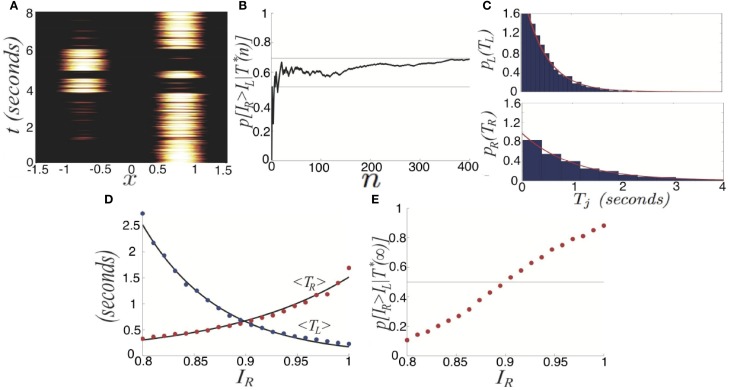
**Purely noise-induced switching in the stochastic neural field (Equation 27). (A)** Single realization of (Equation 27) with asymmetric inputs *I*_*R*_ = 0.92 and *I*_*L*_ = 0.88, leads to longer dominance times for right percept *T*_*R*_. **(B)** Likelihood *p*[*I*_*R*_ > *I*_*R*_|T^*^(*n*)] that the right input *I*_*R*_ is stronger than left *I*_*L*_ based on *n* comparisons of dominance times *T*_*R*_ and *T*_*L*_ sampled. Upper gray line is theoretical prediction (Equation 29) of the limit *n* → ∞. **(C)** Numerically computed dominance time distributions (blue bars) are well fit by the exponential distribution (Equation 28) for the left (〈*T*_*L*_〉 ≈ 0.5 s) and right (〈*T*_*R*_〉 ≈ 1 s) percepts. **(D)** Dependence of mean dominance times 〈*T*_*R*_〉 and 〈*T*_*L*_〉 on the strength of the right input *I*_*R*_ when *I*_*L*_ = 0.9. Black curves are best fits to exponential functions of *I*_*R*_. **(E)** Expected likelihood *p*[*I*_*R*_ > *I*_*L*_ | T^*^(∞)] right input *I*_*R*_ is stronger than left *I*_*L*_ in the limit of high sample number *n* → ∞, as computed theoretically by Equation (29). Other parameters are κ = 0.5, and ε = 0.04.

We explore this further in the case of asymmetric inputs, showing dominance times are still specified by exponential distributions as shown in Figure 6. Despite the fact *I*_*R*_ > *I*_*L*_, the exponential distributions *p*(*T*_*R*_) and *p*(*T*_*L*_) still have substantial overlap, so sampling from these distributions can yield *T*_*R*_ < *T*_*L*_. Using such a sample to guess the ordering of amplitudes *I*_*R*_ and *I*_*L*_ would yield *I*_*R*_ < *I*_*L*_, rather than the correct *I*_*R*_ > *I*_*L*_. In terms of conditional probabilities, we expect situations where *p*(*I*_*R*_ > *I*_*L*_ | T^*^(*n*)) < 1/2 for finite *n*, even though *I*_*R*_ > *I*_*L*_. We can quantify this effect numerically, as shown in Figure 6B. Since the marginal distributions are approximately exponential

(28) $$p_{j}(T_{j})={\text{e}}^{{\text{−T}}_{\text{j}}/\langle {\text{T}}_{j}\rangle }\;/\langle {\text{T}}_{j}\rangle \;\mathrm{j=L},R,$$

we can approximate the conditional probability

(29) $$\begin{array}{l} p[I_{R}>I_{L}|T^{*}(\infty )]=\int_{0}^{\infty }\int_{0}^{x}p_{R}(x)p_{L}(y)\text{d}y\text{d}x\\ \;=\frac{\langle T_{R}\rangle }{\langle T_{R}\rangle +\langle T_{L}\rangle }. \end{array}$$

Using Equation (29), we can estimate the limit *p*(*I*_*R*_ > *I*_*L*_| T^*^(∞)) (Figure 6B). Recent psychophysical experiments suggest humans would perform this task of contrast differentiation of bistable images in this way (Moreno-Bote et al., 2011).

We also see the mean dominance times still obey Levelt's propositions (Figure 6D). Thus, comparing the mean dominance times 〈*T*_*R*_〉 and 〈*T*_*L*_〉 provides very precise information about the ordering of contrasts *I*_*R*_ and *I*_*L*_. However, when comparing successive dominance times, accurately discerning the relative input contrasts is more difficult. This becomes more noticeable when the input contrasts are quite close to one another, as in Figure 6E. We will explore now how introducing depression along with noise improves discernment of the input contrasts by an observer using simple comparison of dominance times.

### Switching through combined depression and noise

**Figure 7:**
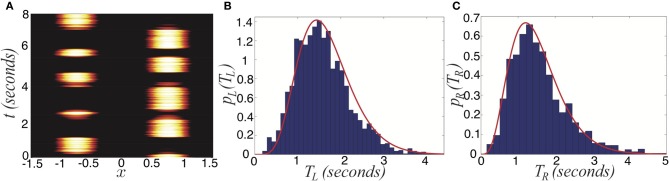
**Switching in the stochastic ring model with depression (Equation 1) with asymmetric inputs (*I*_*a*_ > 0). (A)** Single realization for asymmetric inputs with *I*_*R*_ = 0.92 and *I*_*L*_ = 0.88, which leads to right percept dominating longer. **(B)** Distribution of left percept dominances times *p*_*L*_(*T*_*L*_) over 1000 s is well fit by a gamma distribution (Equation 30). **(C)** Distribution of right percept dominance times *p*_*R*_(*T*_*R*_) across 1000 s is well fit by a gamma distribution (Equation 30). Other parameters are κ = 0.5, β = 0.2, τ = 50, and ε = 0.01.

**Figure 8:**
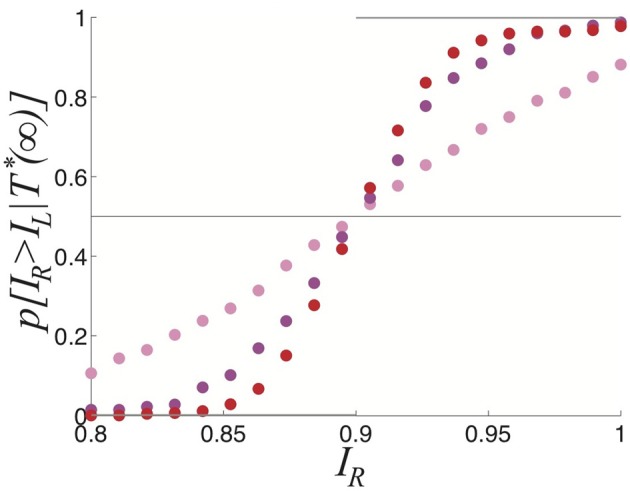
**Comparing the probability densities of dominance times in the stochastic ring model with depression (Equation 1).** Expected likelihood *p*[*I*_*R*_ > *I*_*R*_|T^*^(∞)] the right input *I*_*R*_ is stronger than the left *I*_*L*_ based in the limit of an infinite number of samples of the dominance times *T*_*R*_ and *T*_*R*_ for the parameters: β = 0, ε = 0.04 (pink); β = 0.2, ε = 0.01 (magenta); and β = 0.4 and ε = 0.0025 (red). Other parameters are τ = 50 and κ = 0.5.

We now study the effects of combining noise and depression in the full ring model of perceptual rivalry (Equation 1). Numerical simulations of Equation (1) reveal that noise-induced switches occur robustly, even in parameter regimes where the noise-free system supports no rivalrous oscillations, as shown in Figure 7. Rather than dominance times being distributed exponentially, they roughly follow a gamma distribution (Fox and Herrmann, 1967; Lehky, 1995)

(30) $$p_{j}(T_{j})=\frac{1}{\sigma^{k}\Gamma (k)}T_{j}^{k}\exp\left[-T_{j}/\sigma \right],\;k>1,$$

which is peaked away from zero at *T*_*j*_ = *k*σ, the mean of the distribution. We show two gamma distributions of dominance times with different means can be more easily discerned than two exponential distributions. Gamma distributions with different means are better separated than two exponential distributions. We summarize how this separation improves the inference of relative contrast in Figure 8. As the strength β of depression is increased discernment of relative contrast from sampling dominance time distributions is improved. The likelihood assigned to *I*_*R*_ being greater than *I*_*L*_ is a sigmoidal function of *I*_*R*_ whose steepness increases with β. For no noise, the likelihood function is simply a step function *H*(*I*_*R*_ > *I*_*L*_), implying perfect discernment.

### Analyzing switching in a reduced model

We now perform similar analysis on a reduced network model (Equation 6) and extend some of the results for the ring model. We can construct an energy function (Hopfield, 1984), which provides us with intuition as to the exponential dependence of mean dominance times on input strengths in the noise-driven case. In particular, we analyze Equation (6) where the firing rate function is Heaviside (Equation 4), starting with the case of no noise

(31a) $${\dot{u}}_{R}=-u_{R}+H(I_{R}-q_{L}u_{L}),$$

(31b) $${\dot{u}}_{L}=-u_{L}+H(I_{L}-q_{R}u_{R})$$

(31c) $$\tau {\dot{q}}_{R}=1-q_{R}-\beta u_{R}q_{R},$$

(31d) $$\tau {\dot{q}}_{L}=1-q_{L}-\beta u_{L}q_{L}.$$

First, we note Equation (31) has a stable winner-take-all solution in the *j*th population (*j* = *R, L*) for *I*_*j*_ > 0 and *I*_*k*_ < 1/(1 + β) (*k* ≠ *j*). Second, a stable fusion state exists when both *I*_*L*_, *I*_*R*_ > 1/(1 + β). Coexistent with the fusion state, there may be rivalrous oscillations, as we found in the spatially extended system (Equation 1). To study these, we make a similar fast-slow decomposition of the model (Equation 31), assuming τ » τ_*m*_ to find *u*_*j*_'s possess the quasi-steady state

(32) $$u_{R}=H(I_{R}-q_{L}u_{L}),\;u_{L}=H(I_{L}-q_{R}u_{R}).$$

so we expect *u*_*j*_ = 0 or 1 almost everywhere. Therefore, we can estimate the dominance time of each stimulus using a piecewise equation for the slow subsystem

(33) $$\tau q_{j}=\{\begin{array}{ll} 1-q_{j}-\beta q_{j} & :u_{j}=1,\\ 1-q_{j} & :u_{j}=0, \end{array}\;j=L,R.$$

**Figure 9:**
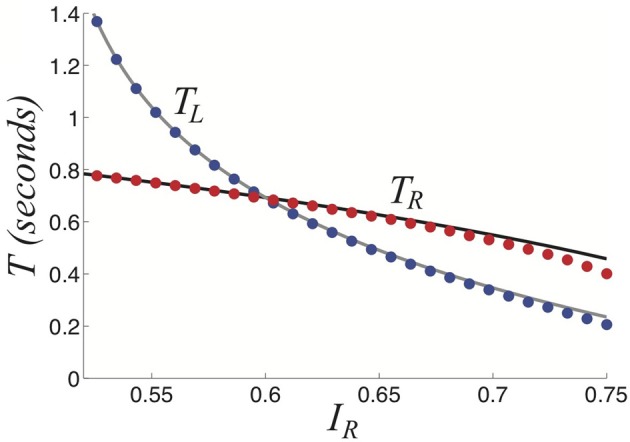
**Dominance times *T*_*L*_ and *T*_*R*_ as a function of right input *I*_*R*_ keeping *I*_*L*_ = 0.8 fixed as computed by theory (curves) in Equations (34) and (35) fits numerically computed (dots) very well.** Other parameters are β = 1 and τ = 50.

Combining the slow subsystem (Equation 33) with the quasi-steady state (Equation 32), we can use self-consistency to solve for the dominance times *T*_*R*_ and *T*_*L*_ of the right and left populations. We simply note that switches will occur through escape, when cross-inhibition is weakened enough by depression such that the suppressed population's (*j*) input becomes superthreshold, so *I*_*j*_ = *q*_*k*_. Using Equation (33), we find

(34) $$T_{R}=\tau \ln\left[\frac{Q_{-}+\sqrt{Q_{-}^{2}-4B_{R}}}{2(1+\beta )I_{L}-2}\right],$$

(35) $$T_{L}=\tau \ln\left[\frac{Q_{+}+\sqrt{Q_{+}^{2}-4B_{L}}}{2(1+\beta )I_{R}-2}\right],$$

where *Q*_±_ = β ± (1+β) [*I*_*R*_ − *I*_*L*_] and *B*_*R, L*_ = (1 − *I*_*R, L*_) (1 + β) [(1 + β) *I*_*L, R*_ − 1]. For symmetric stimuli, *I*_*L*_ = *I*_*R*_ = *I*, both Equations (34) and (35) reduce to

(36) $$T=\tau \ln\left[\frac{\beta +\sqrt{\beta^{2}-4(1-I)(1+\beta )[(1+\beta )I-1]}}{2(1+\beta )I-2}\right]\;,\;$$

using which we can solve for the critical input strength *I* above which only the fusion state exists, *I* = (2 + β)/[2(1+ β)], in the case of symmetric inputs. We show in Figure 9 that this asymptotic approximations Equations (34) and (35) of the dominance times match well with the results of numerical simulations, recapitulating Levelt's propositions.

**Figure 10:**
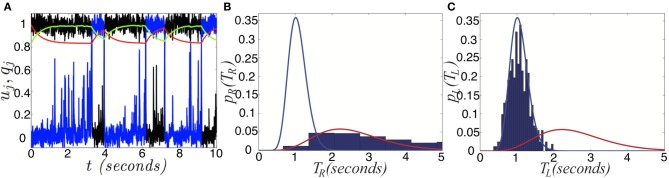
**Switching induced by noise and depression. (A)** Single realization of the network (Equation 6) with depression and noise. Activity variables *u*_*R*_ (black) and *u*_*L*_ (blue) stay close to attractors at 0 and 1, aside from depression or noise induced switching. Depression variables *q*_*R*_ (red) and *q*_*L*_ (green) slowly exponentially change in response to the states of *u*_*R*_ and *u*_*L*_. **(B)** Right and **(C)** left dominance time distributions fit with gamma distributions (Equation 30), in the network (Equation 6) with depression and noise in the case of asymmetric inputs *I*_*R*_ = 0.82 and *I*_*L*_ = 0.78, sampled over 1000 s. The right population has a longer mean dominance time. Other parameters are β = 0.2, τ = 50, and ε = 0.036.

Next, we show that the network with depression and noise generates activity oscillations with dominance times that are gamma distributed (Fox and Herrmann, 1967; Lehky, 1995; Brascamp et al., 2006). We now provide some analytic intuition as to how gamma distributed dominance times may arise in the fast-slow system. First, we display as single realization of the network (Equation 6) in Figure 10A. An approximate energy function for Equation (6) can be computed in the limit of slow depression recovery time τ » τ_*m*_ by assuming we can augment the energy of the depression-free (β = 0) network (Hopfield, 1984)

$$\begin{array}{l} E[u_{R},u_{L}]=H(I_{L}-u_{R})H(I_{R}-u_{L})\\ \;-\;I_{L}H(I_{L}-u_{R})-I_{R}H(I_{R}-u_{L}), \end{array}$$

by the synaptic scalings imposed by *q*_*R*_ and *q*_*L*_ (Mejias et al., 2010), so

$$\begin{array}{l} E[u_{R},u_{L},q_{R},q_{L}]=H(I_{L}-q_{R}u_{R})H(I_{R}-q_{L}u_{L})\\ \;-\;\frac{I_{L}}{q_{R}}H(I_{L}-q_{R}u_{R})-\frac{I_{R}}{q_{L}}H(I_{R}-q_{L}u_{L}). \end{array}$$

A similar energy function was previously used in a model with spike frequency adaptation (Moreno-Bote et al., 2007). Here, we are able to derive the energy function from the model (Equation 6). Therefore, the energy gap between a winner-take-all state and the fusion state will be time-dependent, varying as the synaptic scaling variables *q*_*R*_ and *q*_*L*_ change. The energy difference between the right dominant state and fusion is

$$\Delta E_{R}(t)=1-\frac{I_{L}}{q_{R}(t)},\;\Delta E_{L}(t)=1-\frac{I_{R}}{q_{L}(t)},$$

for the right and left population, respectively.

Notice that dominance times of stochastic switching (Figures 10B,C) in Equation (6) are distributed roughly according to a gamma distribution (Equation 30). Superimposing the probability density of right (left) dominance times on the left (right) probability density, we see they are reasonably separated. Using the analysis we performed for the spatially extended system, we could also show that depression improves discernment of the input contrast difference. Mainly here, we wanted to provide a justification as to the relationship between input strength and mean dominance times. Using energy arguments, we have provided reasoning behind why Levelt's propositions are still preserved in this model, when noise is included, even when switches are noise-induced. Increasing one input leads to a reduction in the energy barrier between the *other* population's winner-take-all state and the fusion state. This leads to the *other* population's dwell time being shorter.

### Switching between three percepts

**Figure 11:**
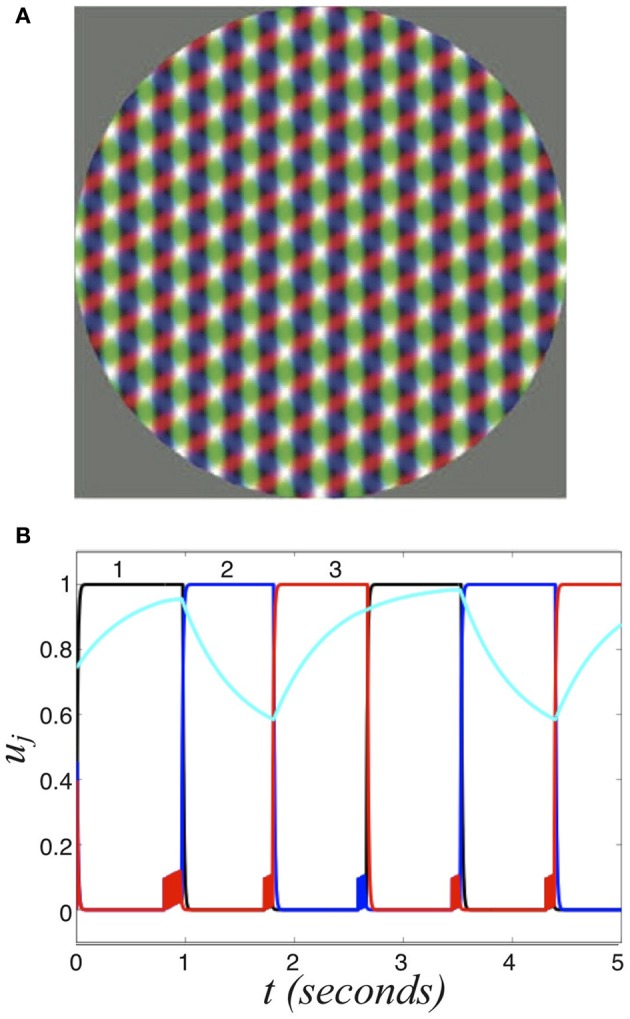
**Perceptual tristability. (A)** Three overlapping grating stimuli, which generates tristable perception. Redrawn with permission from Naber et al. (2010). **(B)** Numerical simulation of Equation (37) showing the activity variables *u*_1_, *u*_2_, *u*_3_ and the second synaptic scaling variable *q*_2_ (cyan) of the three population network (Equation 37) driven by symmetric stimulus *I* = 0.6. Other parameters are β = 1 and τ = 50.

Finally, we will compare the transfer of information in competitive networks that process more than two inputs. Recently, experiments have revealed that perceptual multistability can switch between three or four different percepts (Fisher, 1968; Burton, 2002; Naber et al., 2010; Hupé and Pressnitzer, 2012). In particular, the work of Naber et al. (2010) characterized some of the switching statistics during the oscillations of perceptual tristability. Figure 11A shows an example of a tristable percept. Since dominance times are gamma distributed and there is memory evident in the ordering of percepts (Naber et al., 2010), the process is also likely governed by some slow adaptive process in addition to fluctuations.

We study perceptual tristability in a competitive neural network model with only depression, to start, with a Heaviside firing rate (Equation 4), and symmetric inputs *I*_1_ = *I*_2_ = *I*_3_ = *I*, we study the system

(37a) $${\dot{u}}_{1}=-u_{1}+H(I-q_{2}u_{2}-q_{3}u_{3}),$$

(37b) $${\dot{u}}_{2}=-u_{2}+H(I-q_{1}u_{1}-q_{3}u_{3}),$$

(37c) $${\dot{u}}_{3}=-u_{3}+H(I-q_{1}u_{1}-q_{2}u_{2}),$$

(37d) $$\tau {\dot{q}}_{j}=1-q_{j}-\beta u_{j}q_{j},\;j=1,2,3,$$

**Figure 12:**
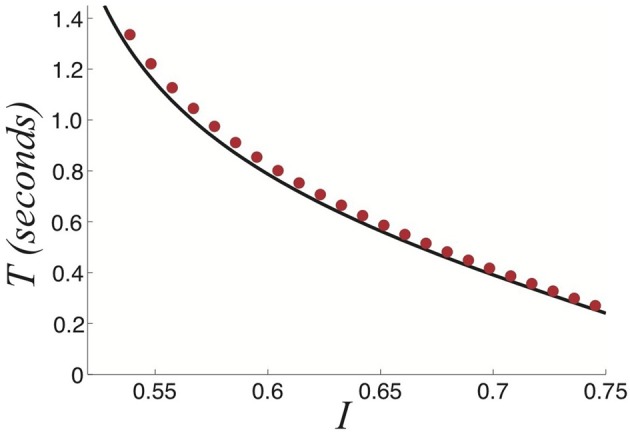
**Relationship between the strength of the stimulus *I* and the dominance times *T* computed using fast-slow analysis (black) and numerics (red dots) for a *perceptually tristable* stimulus.** Other parameters are β = 1 and τ = 50.

We are interested in rivalrous oscillations, which do arise in this network (Figure 11B). Once again, we can perform a fast-slow decomposition of our system, assuming τ » τ_*m*_ to compute the dominance time *T* of a population as it depends on input strength *I*. We find

$$T=\tau \ln\left[\frac{B+\sqrt{B[3I(1+\beta )+\beta -3]}}{2[(1+\beta )I-1]}\right],$$

where *B* = (1 − *I*)(1 + β), which compares very well with numerically computed dominance times in Figure 12. Recent experimental observations have suggested relationships between mean dominance time and input contrast in perceptual tristability may be similar to the two percept case (Hupé and Pressnitzer, 2012). In our model, we see that as the input strength is increased, dominance times decrease. One other important point is that percept dominance occurs in the same order every time (Figure 11B): one, two, three. There are no “switchbacks.” We will show that switchbacks can occur in the noisy regime, which degrades history dependence.

**Figure 13:**
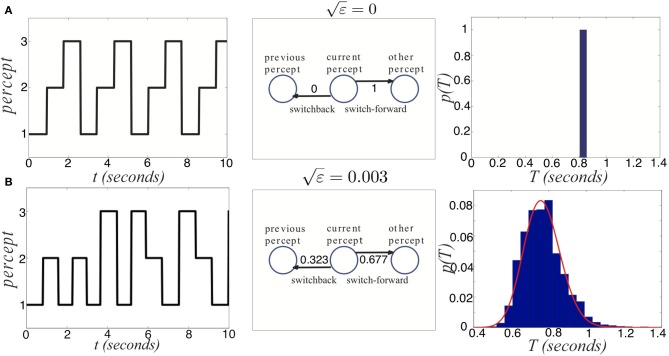
**Noise degrades two sources of information provided by dominance switches. (A)** In the absence of noise, switches always move “forward,” so that the previous percept perfectly predicts the subsequent percept. Dominance times accumulate at a single value too. **(B)** For non-zero noise ($\sqrt{\varepsilon }$ = 0.003), “switch backs” can occur where the subsequent percept is the same as the previous percept. Also, the distribution of dominance times spreads. Other parameters are *I* = 0.6, β = 1, and τ = 50.

**Figure 14:**
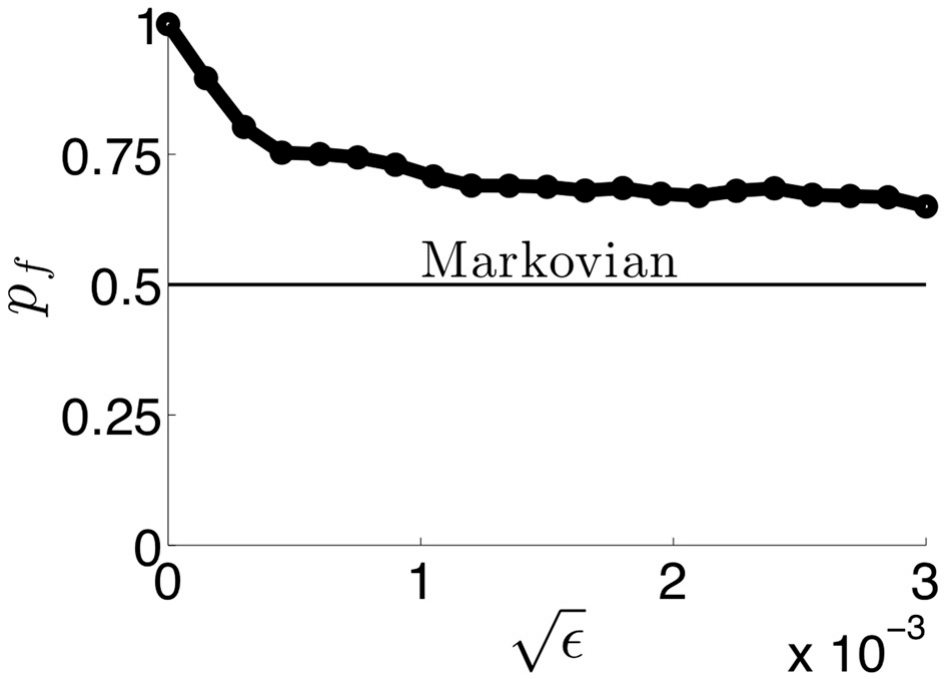
**The probability *p*_*f*_ of a switch being in the forward direction in simulations of (Equation 38) as a function of the amplitude $\sqrt{\varepsilon }$ of noise.** As $\sqrt{\varepsilon }$ increases, network switches behave in more of a Markovian way, not reflecting any memory of the previous percept. Therefore, information of the previous percept is lost as soon as a switch occurs.

Now, we study how noise alters the switching behavior when added to the deterministic network (Equation 37). Thus, we discuss the three population competitive network with noisy in activity

(38a) $${\dot{u}}_{1}=-u_{1}+H(I-q_{2}u_{2}-q_{3}u_{3})+\xi_{1},$$

(38b) $${\dot{u}}_{2}=-u_{2}+H(I-q_{1}u_{1}-q_{3}u_{3})+\xi_{2},$$

(38c) $${\dot{u}}_{3}=-u_{3}+H(I-q_{1}u_{1}-q_{2}u_{2})+\xi_{3},$$

(38d) $$\tau {\dot{q}}_{j}=1-q_{j}-\beta u_{j}q_{j},\;j=1,2,3,$$

where ξ_*j*_ are identical independent white noise processes with variance ε. In Figure 13, we show the noise in Equation (38) degrades two pieces of information carried by dominance switches: the switching time and the direction of switching. Notice that adding noise spreads out the distribution of dominance times (Figure 13B). Thus, there is a less precise characterization of the input strength in the network. Concerning the direction of switching, the introduction of noise makes “switch backs” more likely. We define a “switch back” as a series of three percepts that contains the same percept twice (e.g., 1 → 3 → 1). This is opposed to a “switch forward,” which contains all three percepts (e.g., 1 → 3 → 2). Statistics like these were analyzed from psychophysical experiments of perceptual tristability, using an image like Figure 11A (Naber et al., 2010). The main finding of Naber et al. (2010) concerning this property is that switch forwards occur more often than chance would suggest. Therefore, they proposed that some slow process may be providing a memory of the previous image. Memory in perceptual rivalry has also been observed in experiments where ambiguous stimuli are presented intermittently (Leopold et al., 2002; Pastukhov and Braun, 2008; Gigante et al., 2009). We suggest short term depression as a candidate substrate for this memory. As seen in Figure 13B, the bias in favor of switching forward persists even for non-zero levels of noise. The idea of short term plasticity as a substrate of working memory was also recently proposed in Mongillo et al. (2008). Our results extend this idea, suggesting synaptic mechanisms of working memory may be useful in visual perception tasks, such as understanding ambiguous images. In Figure 14, we show that the process of dominance switching becomes more Markovian, less history dependent, as the level of noise $\sqrt{\varepsilon }$ is increased. In the limit of large noise, the likelihoods of “switch forwards” and “switch backs” are the same, making the ordering of switching purely Markovian.

## Discussion

Mechanisms underlying stochastic switching in perceptual rivalry have been explored in a variety of psychophysical (Fox and Herrmann, 1967; Lehky, 1995; Brascamp et al., 2006), physiological (Leopold and Logothetis, 1996; Blake and Logothetis, 2002), and theoretical studies (Matsuoka, 1984; Laing and Chow, 2002; Moreno-Bote et al., 2007). Since psychophysical data is widely accessible, it can be valuable to use the hallmarks of its statistics as benchmarks for theoretical models. For instance, the fact that dominance time distributions are unimodal functions peaked away from zero suggests that some adaptive process must underlie switching in addition to noise (Laing and Chow, 2002; Brascamp et al., 2006; Shpiro et al., 2009). In addition, Moreno-Bote et al. (2011) information about bistable images may be extracted by sampling a posterior distribution associated with the dominance fraction of each percept. This type of sampling can be well modeled by attractor networks analogous to those presented here (Moreno-Bote et al., 2007). Thus, many dominance time statistics from perceptual rivalry experiments can be employed as points of reference for physiologically based models of visual perception. New data now exists concerning tristable images showing this process also is likely guided by a slow adaptive process in addition to fluctuations (Naber et al., 2010).

We have studied various aspects of competitive neuronal network models of perceptual multistability that include short term synaptic depression. First, we were able to analyze the onset of rivalrous oscillations in a ring model with synaptic depression (York and van Rossum, 2009; Kilpatrick and Bressloff, 2010a). Stimulating the network with a bimodal input leads to winner-take-all solutions, in the form of single bumps, in the absence of synaptic depression. As the strength of synaptic depression is increased, the network undergoes a bifurcation which leads to slow oscillations whose timescale is set by that of synaptic depression. Each stimulus peak is represented in the network by a bump whose dominance time is set by the height of each peak. When noise is added, dominance time histograms obey a gamma distribution. We considered the simple task of an upstream network inferring the relative contrast of stimuli based on partial and whole observations of the dominance time distribution. Thus, we study how well the dominance times (information output) of the network reflect the relative stimulus contrasts (information input). Sampling dominance times better identifies contrast differences when switches are more depression-driven and less noise-driven. Thus, short term depression improves information transfer of networks that process ambiguous images in multiple ways. To our knowledge, no previous studies have explored how sampling dominance time distributions might be used by upstream neurons to infer relative stimulus contrast.

We also used energy methods in reduced models to understand how a combination of noise and depression interact to produce switching. Using the energy function derived by Hopfield (1984) for analog neural networks, we justify the exponential dependence of dominance times upon input strength in purely noise-driven switching. Studying an adiabatically derived energy function for the case of slow depression, we also show how depression works to reduce the energy barrier between winner-take-all states, leading to the slow timescale that defines the peak in depression-noise generated switches. Finally, using a three population space-clamped neural network, we analyzed depression and noise generated switching that may underlie perceptual tristability. We found this network also sustained some of the same relationships between input contrast and dominance times as the two population network. When switches are generated by depression there is an ordering to the population dominance that is lost when switches are noise generated. This is due to the memory generated by short term depression (Mongillo et al., 2008), so the switching process is non-Markovian due to the inherent slow timescale in the background. Dynamical variability must be weak enough to not totally wash out the non-Markovian character of switches. To our knowledge, neither short term depression or adaptation has been proposed before as a mechanism for history dependence in the switching between tristable stimuli. Also, no previous authors have used the history dependence of switching observed in Naber et al. (2010) as a bench mark for a perceptually tristable network model. As opposed to tristability, perceptual bistability generally does not demonstrate strong history dependence in dominance time statistics, behaving more as a renewal process (Lehky, 1995; Laing and Chow, 2002). However, there is some recent evidence that suggests there may be very minor serial correlations in dominance times (van Ee, 2009), likely arising as a signature of a slow adaptive process partially responsible for switching.

Mutual inhibitory rate models with terms representing only spike frequency adaptation (Wilson, 2003; Moreno-Bote et al., 2007) or only short term depression (Kilpatrick and Bressloff, 2010b; Bressloff and Webber, 2012) or both adaptation and depression (Laing and Chow, 2002; Shpiro et al., 2007; Seely and Chow, 2011) have been analyzed in several previous studies. Both mechanisms, when they are included in rate models, can generate dominance time statistics that correspond well with the stimulus contrast dependencies of Levelt (1965), if placed in the right parameter regime. One subtle difference is that if the firing rate function is steep enough in models with depression only, there are no parameter regimes where dominance times increase with contrast (Seely and Chow, 2011). Even if the firing rate function is not very steep, rate models with only depression favor parameter regimes where dominance times decrease with contrast. The effect is not seen in mutually inhibitory rate models with only adaptation (Shpiro et al., 2007). Since Levelt (1965) observed that dominance times decrease with contrast, this suggests depression may be a more suitable choice of slow negative feedback in models of perceptual multistability. On the other hand, it has been demonstrated that gamma distributed dominance time distributions also emerge in perceptual rivalry models with spike frequency adaptation (Shpiro et al., 2009), so it seems the models may often yield similar results (see Shpiro et al., 2007). Note, we have demonstrated a combination of mutual inhibition and depression can generate ordered switching that may be a substrate of perceptual tristability. We presume these results would also extend to a model with mutual inhibition and spike frequency adaptation.

Spatially extended neural field models are a useful tool for understanding complex dynamics that emerge in networks connected by synapses that are stimulus preference dependent (Wilson and Cowan, 1973; Amari, 1977; Bressloff and Cowan, 2002). Processes underlying perceptual rivalry can evolve with a characteristic spatiotemporal structure, as has been found in experiments where observers report waves of visual dominance sweeping one percept over another (Wilson et al., 2001). Bressloff and Webber (2012) and Webber and Bressloff (2013) recently modeled this using a two spatially extended populations coupled to one another by mutual inhibition, where short term depression leads to switches in the direction of activity wave propagation. Our work is distinct from this in several ways. First, we are concerned with non-propagating activity whose switches are abrupt, not gradual as in Bressloff and Webber (2012). In addition, we compute dominance time distributions whereas Bressloff and Webber (2012) compute mean first passage time distributions for their traveling wave. Finally, we have demonstrated phenomena that only require a single cortical layer, and their results require one layer for each percept.

Note to analytically study the relationship between dominance times and input contrast in the noisy system, we resorted to a simple space-clamped neural network. In future work, we plan to develop energy methods for spatially extended systems like Equation (27). Such methods have seen success in analyzing stochastic partial differential equation models such as Ginzburg-Landau models (E et al., 2004). Energy functions have recently been developed for neural field models, but have mostly been studied as a means of determining global stability in deterministic systems (Wu et al., 2002). The fact that pure noise does lead to exponentially distributed dominance times suggests it may be possible to develop a large deviations theory for switching in the system (Equation 27), using techniques like those of E et al. (2004). We propose that by deriving the specific potential energy of spatially extended neural fields, it may be possible to approximate the transition rates of solutions from the vicinity of one attractor to another. In the system (Equation 27), there should be some separatrix between the two winner-take-all states that must be crossed in order for a transition to occur. The least action principle states that there is even a specific point on this separatrix through which the dynamics most likely flows (E et al., 2004). Finding this point using an energy function would allow us to relate the parameters of the model to the distribution of dominance times. This would provide a theoretical framework for interpreting data concerning rivalry of spatially extended images, such as those that produce waves (Wilson et al., 2001). We could also extend this work to analyze interocular grouping Lee and Blake (2004), the phenomenon by which partial images split between either eye are grouped together in perception and rival. Thus, we would need to consider several orientation columns associated with each eye. Columns driven by similarly oriented stimuli would excite one another, overriding weak inhibition between columns in different eyes. Our fast-slow analysis could be useful for analyzing how system dynamics might collapse to group images together in perception.

### Conflict of interest statement

The author declares that the research was conducted in the absence of any commercial or financial relationships that could be construed as a potential conflict of interest.
